# Prognostic value of myocardial computed tomography–derived extracellular volume in severe aortic stenosis requiring aortic valve replacement: a systematic review and meta-analysis

**DOI:** 10.1093/ehjci/jeae324

**Published:** 2025-01-10

**Authors:** Andrea Faggiano, Elisa Gherbesi, Stefano Carugo, Matteo Brusamolino, Dan Alexandru Cozac, Elena Cozza, Maria Teresa Savo, Francesco Cannata, Marco Guglielmo, Lucia La Mura, Fabio Fazzari, Nazario Carrabba, Edoardo Conte, Saima Mushtaq, Andrea Baggiano, Andrea Igoren Guaricci, Roberto Pedrinelli, Ciro Indolfi, Gianfranco Sinagra, Pasquale Perrone Filardi, Valeria Pergola, Gianluca Pontone

**Affiliations:** Department of Cardio-Thoracic-Vascular Diseases, Foundation IRCCS Ca’ Granda Ospedale Maggiore Policlinico, 20122 Milan, Italy; Department of Clinical Sciences and Community Health, University of Milan, 20122 Milan, Italy; Department of Cardio-Thoracic-Vascular Diseases, Foundation IRCCS Ca’ Granda Ospedale Maggiore Policlinico, 20122 Milan, Italy; Department of Cardio-Thoracic-Vascular Diseases, Foundation IRCCS Ca’ Granda Ospedale Maggiore Policlinico, 20122 Milan, Italy; Department of Clinical Sciences and Community Health, University of Milan, 20122 Milan, Italy; Department of Clinical Sciences and Community Health, University of Milan, 20122 Milan, Italy; Department of Perioperative Cardiology and Cardiovascular Imaging, Centro Cardiologico Monzino IRCCS, Via C. Parea 4, 20138 Milan, Italy; Cardiology Clinic, Department of Cardiac, Thoracic, Vascular Sciences and Public Health, University of Padova, Padova, Italy; Department of Physiology, University of Medicine, Pharmacy, Science and Technology ‘George Emil Palade’ of Târgu Mureș, 540142 Târgu Mureș, Romania; Cardiology Clinic, Department of Cardiac, Thoracic, Vascular Sciences and Public Health, University of Padova, Padova, Italy; Cardiology Clinic, Department of Cardiac, Thoracic, Vascular Sciences and Public Health, University of Padova, Padova, Italy; Department of Perioperative Cardiology and Cardiovascular Imaging, Centro Cardiologico Monzino IRCCS, Via C. Parea 4, 20138 Milan, Italy; Division of Heart and Lungs, Department of Cardiology, Utrecht University Medical Center, Utrecht University, Utrecht, The Netherlands; Department of Cardiology, Haga Teaching Hospital, The Hague, The Netherlands; Department of Advanced Biomedical Sciences, University Federico II of Naples, Naples, Italy; Department of Perioperative Cardiology and Cardiovascular Imaging, Centro Cardiologico Monzino IRCCS, Via C. Parea 4, 20138 Milan, Italy; Department of Cardiothoracovascular Medicine, Azienda Ospedaliero-Universitaria Careggi, Florence, Italy; Department of Clinical Cardiology and Cardiovascular Imaging, Galeazzi-Sant’Ambrogio Hospital IRCCS, Milan, Italy; Department of Perioperative Cardiology and Cardiovascular Imaging, Centro Cardiologico Monzino IRCCS, Via C. Parea 4, 20138 Milan, Italy; Department of Perioperative Cardiology and Cardiovascular Imaging, Centro Cardiologico Monzino IRCCS, Via C. Parea 4, 20138 Milan, Italy; University Cardiology Unit, Interdisciplinary Department of Medicine, University of Bari Aldo Moro, Bari, Italy; Cardiac, Thoracic and Vascular Department, University of Pisa, Pisa, Italy; Istituto di Cardiologia, Dipartimento di Scienze Mediche e Chirurgiche, Università degli Studi ‘Magna Graecia’, Catanzaro, Italy; Cardiology Specialty School, University of Trieste, Trieste, Italy; Center for Diagnosis and Treatment of Cardiomyopathies, Cardiovascular Department, Azienda Sanitaria Universitaria Giuliano-Isontina (ASUGI), Trieste, Italy; Department of Advanced Biomedical Sciences, University Federico II of Naples, Naples, Italy; Cardiology Clinic, Department of Cardiac, Thoracic, Vascular Sciences and Public Health, University of Padova, Padova, Italy; Department of Perioperative Cardiology and Cardiovascular Imaging, Centro Cardiologico Monzino IRCCS, Via C. Parea 4, 20138 Milan, Italy; Department of Biomedical, Surgical and Dental Sciences, University of Milan, Milan, Italy

**Keywords:** aortic stenosis, aortic valve replacement, computed tomography–derived extracellular volume fraction, cardiac computed tomography angiography

## Abstract

Computed tomography (CT)-derived extracellular volume (ECV) fraction is a non-invasive method to quantify myocardial fibrosis. Evaluating CT-ECV during aortic valve replacement (AVR) planning CT in severe aortic stenosis (AS) may aid prognostic stratification. This meta-analysis evaluated the prognostic significance of CT-ECV in severe AS necessitating AVR. Electronic database searches of PubMed, Ovid MEDLINE, and Cochrane Library were performed. The primary outcome was to compare the occurrence of a composite of cardiovascular outcomes in patients with severe AS undergoing AVR with elevated myocardial CT-ECV values vs. patients with normal values. Secondary outcomes included all-cause mortality and heart failure (HF)-related hospitalization. A total of 1223 patients undergoing AVR for severe AS were included in 10 studies: 524 patients with high values of CT-ECV and 699 with normal values of CT-ECV. The pooled CT-ECV cut-off to define elevated values and predict prognosis was 30.7% [95% confidence interval (CI): 28.5–33.7%]. At a mean follow-up of 17.9 ± 2.3 months after AVR, patients with elevated CT-ECV experienced a significantly higher number of cardiovascular events [43.4 vs. 14.0%; odds ratio (OR): 4.3, 95% CI: 3.192–5.764, *P* < 0.001]. Regarding secondary outcomes, all-cause mortality occurred in 29.3% of patients with elevated CT-ECV vs. 11.6% with CT-ECV below the cut-off (OR: 3.5, 95% CI: 2.276–5.311, *P* < 0.001), whereas HF hospitalization was observed in 25.5% vs. 5.9% (OR: 4.9, 95% CI: 2.283–10.376, *P* < 0.001). Patients undergoing AVR for severe AS with elevated CT-ECV values experience a worse post-intervention prognosis. The implementation of CT-ECV evaluation in routine AVR planning protocols should be considered.

## Graphical Abstract

**Graphical Abstract:**
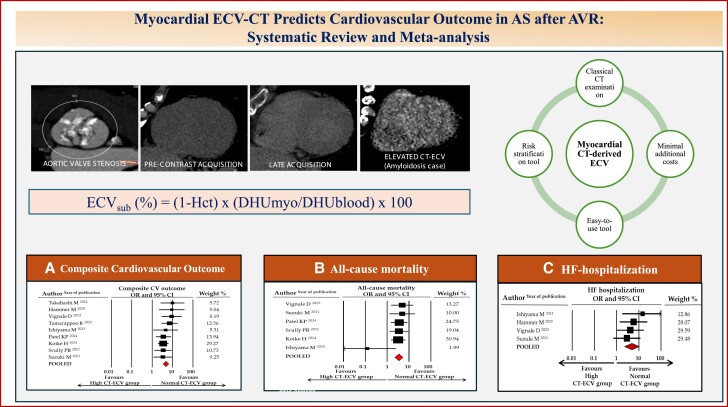

**Clinical Question**: Could CT-ECV predict prognosis in patients with severe aortic stenosis undergoing aortic valve replacement?

**What is the main finding?** Patients undergoing aortic valve replacement for severe aortic stenosis and presenting elevated CT-ECV values experience a worse medium-term post-intervention prognosis compared with those with normal CT-ECV values.

## Introduction

Aortic stenosis (AS) represents the most common valvular heart disease in Europe and North America, and its prevalence is expected to increase due to the ageing population.^1^ Despite major advancements in treatment strategies over the last decades, AS still poses a significant mortality and morbidity burden even in patients undergoing aortic valve replacement (AVR).^2,3^ AS progression induces left ventricular hypertrophy and subsequently myocardial fibrosis, both conditions being associated with high mortality rates.^4^ However, both transcatheter AVR (TAVR) and surgical AVR (SAVR) have been shown to reduce left ventricular mass (LVM) and improve outcomes in patients with AS.^5^ Cardiac computed tomography angiography (CCTA) is the imaging tool of choice for the pre-procedural planning of AVR; it provides key information about the anatomy of the valve and vascular access.^1^ In the last few years, it has been found that CCTA could offer additional interesting information. Cardiac computed tomography–derived extracellular volume (CT-ECV) fraction has emerged as a novel imaging tool, offering insights into myocardial tissue characteristics in response to haemodynamic stress.^6^ Cardiovascular magnetic resonance (CMR) with gadolinium contrast is currently regarded as the gold standard for assessing ECV, which consists of both vascular and interstitial spaces.^7^ In a healthy heart, ECV typically ranges from 23 to 28% of myocardial volume.^8^ However, in patients with cardiac diseases, ECV may increase due to interstitial expansion, often resulting from fibrosis, which can negatively impact prognosis.^9^ Nacif *et al.*^10^ have shown that ECV measurements obtained through cardiac CT are both accurate and reliable when compared with CMR. Subsequent studies have consistently demonstrated strong agreement between CMR-derived ECV and CT-ECV across different cardiomyopathies.^6,11^ Since elevated ECV is a hallmark of cardiac amyloidosis, incorporating CT-ECV measurements into cardiac CT protocols—such as during pre-TAVR evaluations—could facilitate the identification of the estimated 15% of patients with coexisting transthyretin amyloidosis.^12^ Importantly, this integration would have minimal effects on radiation exposure or scan duration. However, although CT-ECV is known to correlate with myocardial fibrosis, its effect on cardiovascular outcomes in patients with AS who undergo AVR is still unclear. This study seeks to fill this knowledge gap by evaluating the prognostic value of CT-ECV in patients with severe AS. Therefore, the present systematic review and meta-analysis aim to explore the prognostic significance of CT-ECV in severe AS necessitating AVR, seeking to elucidate its potential as a predictive tool for enhancing patient risk stratification and guiding clinical decision-making in this vulnerable population.

## Methods

The present research was performed following the Preferred Reporting Items for Systematic Reviews and Meta-Analyses (PRISMA) guidelines.^13^ Additionally, it followed the specific guidelines for prognostic meta-analyses^14^ and was registered on the International Prospective Register of Systematic Reviews ‘PROSPERO’ under the identifier: CRD42024539439. Pertinent literature was systematically scrutinized to identify all studies assessing how myocardial CT-ECV values impact any cardiovascular outcomes post-AVR in individuals with severe AS. The PubMed, Ovid MEDLINE, and Cochrane Library databases were analysed to search English-language papers published from the inception up to 20 October 2024. Studies were identified by using Me-SH terms and crossing the following terms: ‘myocardial extracellular volume’, ‘myocardial extracellular volume quantification’, ‘myocardial extracellular volume quantification fraction’, ‘computed tomography’, and ‘cardiac computed tomography’. An artificial intelligence tool (*Consensus*: https://consensus.app/) was used to verify whether any studies had been missed by the traditional search. The main inclusion criteria were: (i) English original papers, research letters, and short communications published in peer-reviewed journals; no unpublished or grey literature was searched; (ii) studies assessing myocardial CT-ECV in patients with severe AS prior to undergoing AVR; (iii) studies investigating the association between myocardial CT-ECV values before AVR and subsequent post-intervention cardiovascular outcomes (no specific cardiovascular outcomes were predetermined beforehand); (iv) studies comparing post-intervention cardiovascular outcomes between patients with presumed elevated CT-ECV values (no specific cut-off was defined beforehand) and those with normal values; and (v) inclusion of essential population clinical and demographic characteristics. Specific exclusion criteria were: (i) studies with <10 patients, no predetermined maximum sample size was set; (ii) studies reporting myocardial CT-ECV data before AVR but lacking information on post-intervention cardiovascular outcomes; (iii) narrative/systematic reviews, editorials, abstracts, and case reports were excluded from analyses (but examined for potential additional references).

Literature search and data extraction were performed by four reviewers (M.B., M.T.S., El.C., and D.A.C.) and independently checked by another reviewer (A.F.) that resolved disagreements on study judgements. For each eligible study, data including article information (first author and year of publication), study characteristics (study design, number of patients, and follow-up time), relevant population demographics [age, gender, diabetes, hypertension, dyslipidaemia, smoking, previous myocardial infarction (MI), percutaneous coronary intervention, coronary artery bypass graft], echocardiographic and CT specifics, as well as clinical outcomes of interest were systematically extracted, aligning with recommended standards of CHARMS-PF checklist. Where essential data were not available, they were requested from the specific corresponding author and, if it was not possible to obtain them, they were extrapolated from the figures of the studies (when the scale and resolution of the figures were sufficiently precise to allow accurate extrapolation of data). For the study of Hammer *et al.*,^15^ the comparative data between high and normal CT-ECV patients (with the specific cut-off identified by the authors in the published manuscript) were obtained by analysing the original database provided by the authors in Supplementary material. In cases where extreme asymmetry of the confidence interval (CI) precluded meta-analysis, the upper limit of the CI was conservatively adjusted downward, with the sole possibility that the effect size obtained may be underestimated compared with the real value (not objectively observable due to meta-analytic constraints). When quantitative variables were expressed as median and CI, the mean and standard deviation required for meta-analysis were derived using specific appropriate formulas.^16,17^ The QUIPS tool for assessing the quality of prognostic studies in meta-analyses was used.^18^ All analyses were based on previously published studies; thus, no ethical approval or patient consent was required.

The primary outcome of the meta-analysis was to compare the cardiovascular outcome (provided by each study, albeit different) in patients with severe AS undergoing AVR with elevated myocardial CT-ECV values (with cut-off provided by each study, albeit different) vs. patients with normal values. Secondary outcomes included all-cause mortality and hospitalization due to heart failure (HF). To this purpose, a pooled analysis of these parameters was performed using fixed or random effects models by Comprehensive Meta-Analysis Version 2 (Biostat, Englewood, NJ, USA). Heterogeneity was estimated by using *I*^2^, *Q*, and *τ*^2^ values; random or fixed effect models were applied according to heterogeneity across studies (*I*^2^).^19^ Standardized mean difference (SMD) and odds ratios (ORs) with 95% CI were calculated to evaluate the statistical difference of variables and outcomes in elevated and normal CT-ECV groups. The limit of statistical significance was set at *P* < 0.05.

We used the forest plot to graphically present the results of each included study: the studies are ordered by OR values, from highest to lowest, to facilitate comparison of effect sizes and highlight studies with the most pronounced effects at the top.

Publication bias was assessed by using the funnel plot method according to the trim and fill test. Observed and adjusted values, and their lower and upper limits, have been calculated. To assess the effect of individual studies on the pooled result, we conducted a sensitivity analysis by excluding each study one by one and recalculating the combined estimates on the remaining studies. The PRISMA 2020 statement, a 27-item checklist addressing the introduction, methods, results, and discussion sections of a systematic review report, is available in the Supplementary material.

## Results

### Characteristics of the included studies

**Figure 1:**
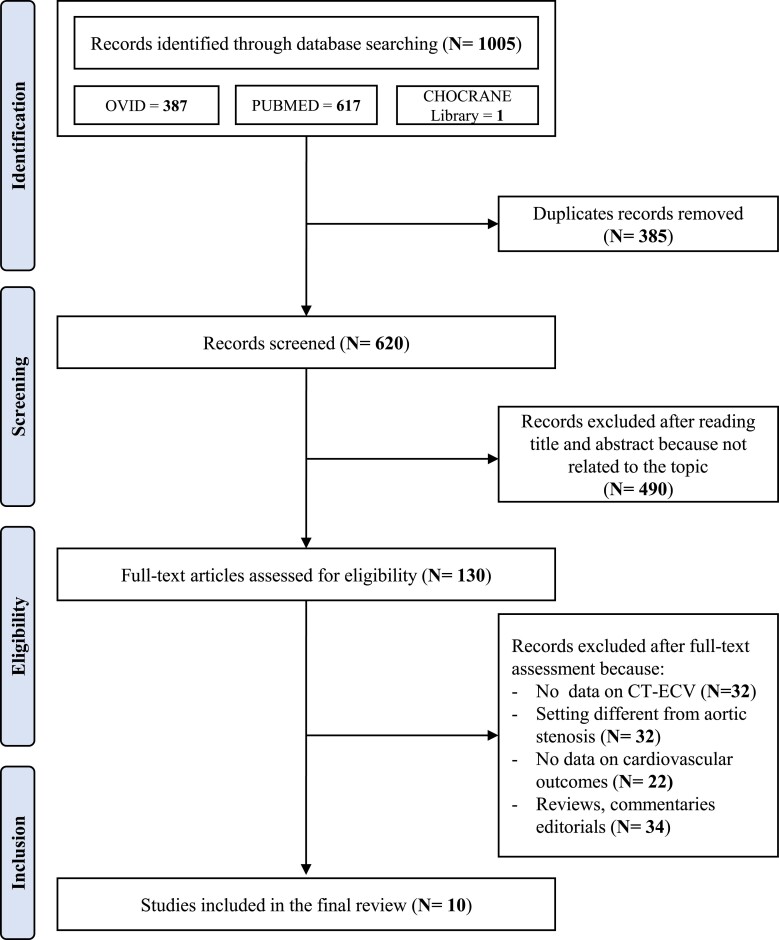
PRISMA flow chart showing the search strategy and manuscripts selection process.

After removing duplicates, the initial literature search identified 620 papers. The PRISMA flow chart showing the search strategy and manuscript selection process is illustrated in *Figure 1*.

After the initial screening of titles and abstracts, 490 studies were excluded as they were not related to the topic. Therefore, 130 studies were reviewed; of these, 32 did not report data on myocardial CT-ECV, 22 did not report data on cardiovascular outcomes after AVR, 32 referred to clinical settings different from AS, and 34 were reviews, commentaries, and editorials. According to the QUIPS tool, the included studies showed a low or moderate risk of bias across the six key domains. Therefore, no study was excluded based on limited quality.

A total of 1223 patients undergoing AVR for severe AS were included in 10 studies (sample size ranging from 57 to 300) performed in 3 continental areas (Europe = 3, Asia = 4, America = 3): 524 patients with high values of CT-ECV and 699 with normal values of CT-ECV.^15,20–28^

*Table 1* summarizes the main findings of selected studies, such as authors, year of publication, study design, AS definition and typology, AVR procedures, and cardiovascular outcomes. All the included studies defined severe AS in accordance with international guidelines (aortic valve area <1 cm^2^, indexed aortic valve area <0.6 cm^2^/m^2^, mean gradient >40 mmHg, max velocity >4 m/s).^1,29^ Whereas one study exclusively enrolled subjects with low-flow, low-gradient severe AS, the remaining studies generally had a low prevalence of this condition. Notably, and better discussed in the Limitations section, only two studies (Scully *et al.*^23^ and Patel *et al.*^28^) opportunistically excluded the presence of cardiac amyloidosis, whereas another study [Koike *et al*.^26^] excluded subjects with a clinical history of cardiac amyloidosis. The majority of studies (8 out of 10) involved only patients undergoing TAVR, with only 2 studies allowing for SAVR as an alternative, albeit with a higher proportion of patients allocated to TAVR over SAVR. Despite the heterogeneity of cardiovascular outcomes across the included studies, the majority of them (6 out of 10) utilized the same composite outcome, comprising HF hospitalization and all-cause mortality. Three studies adopted either HF hospitalization or all-cause mortality as their outcome measures, whereas Han *et al.*^21^ stand out as the only study to employ a clinically different cardiovascular outcome, namely left ventricular ejection fraction (LVEF) recovery.

**Table 1:** Main general characteristics of included studies

First author, year	Country	Study design	Sample size	Cardiac amyloidosis excluded	AS definition	LF-LG AS *n* (%)	Primary outcome	Secondary outcome (Y/N)		AVR (%)
								All-cause mortality	HF hospitalization	
Hammer M, 2021^15^	Asia (Israel)	Prospective single centre	57	No	AHA/ESC guidelines	NA	HF hospitalization + stroke	No	Yes	TAVR (88%), SAVR (12%)
Tamarappoo B, 2020^20^	America (USA)	Retrospective single centre	150	No	AHA/ESC guidelines	150 (100%)	HF hospitalization + all-cause mortality	No	No	TAVR (100%)
Han D, 2021^21^	America (USA)	Retrospective single centre	109	No	AHA/ESC guidelines	NA	LVEF recovery (absolute increase of LVEF ≥ 10%)	No	No	TAVR (100%)
Suzuki M, 2021^22^	Asia (Japan)	Retrospective single centre	95	No	AHA/ESC guidelines	9 (9%)	HF hospitalization + all-cause mortality	Yes	Yes	TAVR (78%), SAVR (22%)
Scully PR, 2022^23^	Europe (UK)	Retrospective single centre	106	Yes	AHA/ESC guidelines	NA	All-cause mortality	Yes	No	TAVR (100%)
Ishiyama M, 2023^24^	Asia (Japan)	Retrospective single centre	71	No	AHA/ESC guidelines	14 (19.7%)	HF hospitalization + all-cause mortality	Yes	Yes	TAVR (100%)
Vignale D, 2023^25^	Europe (Italy)	Prospective single centre	113	No	AHA/ESC guidelines	15 (13%)	HF hospitalization + all-cause mortality	Yes	Yes	TAVR (100%)
Koike H, 2024^26^	America (USA)	Retrospective single centre	300	No (1 patient excluded for prior history of CA)	AHA/ESC guidelines	NA	HF hospitalization + all-cause mortality	Yes	No	TAVR (100%)
Takahashi M, 2024^27^	Asia (Japan)	Retrospective single centre	127	No	AHA/ESC guidelines	6 (5%)	HF hospitalization + all-cause mortality	No	No	TAVR (100%)
Patel KP, 2024^28^	Europe (UK)	Prospective single centre	95	Yes	AHA/ESC guidelines	NA	All-cause mortality	Yes	No	TAVR (100%)

LF-LG, low flow, low gradient; AS, aortic stenosis; AVR, aortic valve replacement; HF, heart failure; ASE, American Society of Echocardiography; ESC, European Society of Cardiology; TAVR, transcatheter aortic valve replacement; SAVR, surgical aortic valve replacement; LVEF, left ventricular ejection fraction; CA, cardiac amyloidosis; NA, not available.

*Table 2* and Supplementary data online, *Table S1* provide a summary of the CT evaluation specifics (including contrast doses) and outlines the process used by selected studies to determine CT-ECV cut-off values. In all included studies, patients underwent CT as part of the routine AVR planning protocol. Additionally, alongside the routine protocol (pre-contrast and arterial phase), a 3–7 min delayed acquisition was conducted to evaluate CT-ECV in all the studies. Most of the included studies (8 out of 10) employed a Siemens Healthcare CT scanner, with 5 studies utilizing the SOMATOM Force model and 3 studies utilizing the SOMATOM Definition Flash model. Details regarding the slice system of the multidetector were specified in only two studies.^15,27^ All studies except one^22^ utilized the ‘subtraction’ methodology for CT-ECV assessment. The majority of studies (8 out of 10) evaluated the mean CT-ECV (referring to the average value across the entire myocardium), whereas 5 also assessed septal CT-ECV. Tamarappoo *et al.*^20^ additionally examined lateral CT-ECV, and only Patel *et al.*^28^ evaluated all segments (anterior, septal, lateral, and inferior) from base to apex, distinguishing between subepicardial and subendocardial regions. Patel *et al.*^28^ was the only group to use subepicardial CT-ECV for defining the cut-off in outcome analysis, six studies used mean CT-ECV whereas three used septal CT-ECV. Four studies determined the ‘high CT-ECV’ cut-off by selecting the value with the best performance on the receiver operating characteristic (ROC) curve to predict outcomes in their respective populations. Conversely, four studies used the median value, whereas two studies used the third quartile of distribution.

**Table 2:** CT specifics and CT-ECV protocols of the studies included in the systematic review and meta-analysis

First author, year	CT scanner	ECV used for outcome	Timing of late acquisition (min)	Myocardial ECV measurement method	ECV cut-off identification method	ECV cut-off (%)
Hammer M, 2021^15^	Brilliance iCT, Philips Healthcare (256-slice system)	Septal ECV	7	ECV subtraction Infarcted segments excluded	ROC curve	40.8
Tamarappoo B, 2020^20^	SOMATOM Definition Flash, Siemens Healthcare	Mean ECV	5	ECV subtraction Infarcted segments excluded	ROC curve	33
Han D, 2021^21^	SOMATOM Definition Flash; Siemens Healthcare	Mean ECV	5	ECV subtraction Infarcted segments excluded	ROC curve	30
Suzuki M, 2021^22^	SOMATOM Force, Siemens Healthcare	Mean ECV	5	ECV iodine	Median ECV	27.8
Scully PR, 2022^23^	SOMATOM Force, Siemens Healthcare	Mean ECV	3/5	ECV subtraction	3rd quartile ECV	29.7
Ishiyama M, 2023^24^	SOMATOM Force, Siemens Healthcare	Mean ECV	5	ECV subtraction	Median ECV	32
Vignale D, 2023^25^	SOMATOM Definition Flash, Siemens Healthcare	Septal ECV	5	ECV subtraction Infarcted segments excluded	3rd quartile ECV	31.3
Koike H, 2024^26^	SOMATOM Force, Siemens Healthcare	Septal ECV	3	ECV subtraction Infarcted segments excluded	Median ECV	28.5
Takahashi M, 2024^27^	Aquilion One/ViSION Edition, Canon Medical Systems (320-row multidetector CT) or Revolution CT Apex, GE Healthcare (256-row multidetector CT)	Mean ECV	6	ECV subtraction	ROC curve	32.6
Patel KP, 2024^28^	SOMATOM Force, Siemens Healthcare	Epicardial ECV	3	ECV subtraction If infarcted segments, patients were excluded	Median ECV	27.1

ECV, extracellular volume; CT, computed tomography; ECV subtraction, subtraction-derived method; ECV iodine, iodine density–derived method; ROC, receiver-operating characteristic.

### Differences between patients with high and normal CT-ECV values

*Table 3* provides an overview of the clinical, echocardiographic, and CT differences between patients with CT-ECV levels above and below the specific cut-off identified in each single study. The pooled CT-ECV cut-off value, derived from the meta-analysis of 10 studies, to define high ECV and predict prognosis, stands at 30.7% (95% CI: 28.5–33.7%). *Table 4* delineates the clinical, echocardiographic, and CT disparities between the two pooled groups categorized as ‘high’ vs. ‘normal’ CT-ECV. Notably, the two pooled groups exhibit homogeneity in terms of age, sex, body mass index, renal function, and arterial hypertension. However, those with high CT-ECV display significantly higher prevalence rates of cardiovascular risk factors (dyslipidaemia and diabetes) and cardiovascular diseases (atrial fibrillation, MI, and percutaneous revascularization). Additionally, they demonstrate significantly diminished LVEF, paralleled by heightened biomarker activity reflected in elevated brain natriuretic peptide levels. Conversely, there are no notable distinctions in the pure severity of aortic valve pathology between the two groups, except for a trend towards marginally lower aortic valve mean gradients among individuals with high CT-ECV.

**Table 3:** Summary of clinical and imaging differences between high CT-ECV patients and normal CT-ECV across all single studies included in the systematic review and meta-analysis

Author, publication year	Sample size (*n*)		Female (%)		Age (years)		Follow-up (months)		CT-ECV (%)		AVAi (cm/m^2^)		Mean gradient (mmHg)		LVEF (%)	
	High CT-ECV	Controls	High CT-ECV	Controls	High CT-ECV	Controls	High CT-ECV	Controls	High CT-ECV	Controls	High CT-ECV	Controls	High CT-ECV	Controls	High CT-ECV	Controls
Hammer M, 2021^15^	26	31	35	65	78.3 ± 7.5	79.6 ± 6.6	12 ± 0	12 ± 0	51.0 ± 8.8	31.4 ± 7.2	0.35 ± 0.13	0.39 ± 0.19	42.4 ± 16.4	55.4 ± 18.7	52.4 ± 8.3	61.2 ± 5.9
Tamarappoo B, 2020^20^	57	93	37	42	81 ± 10	81 ± 9	13.9 ± 5.4	13.9 ± 5.4	40.1 ± 6	25.6 ± 4.6	0.44 ± 0.13	0.45 ± 0.19	25.2 ± 8.3	28.8 ± 7	44.9 ± 18	54 ± 17.6
Han D, 2021^21^	62	47	NA	NA	NA	NA	1.5 ± 0.5	1.5 ± 0.5	NA	NA	NA	NA	NA	NA	31.2 ± 10	33.7 ± 10.2
Suzuki M, 2021^21^	48	47	75	74	83.5 ± 5.6	84.5 ± 4.3	31.2 ± 12.6	31.2 ± 12.6	30.9 ± 2.8	25.2 ± 2.0	0.42 ± 0.13	0.44 ± 0.1	51.4 ± 13.8	51.5 ± 13.2	61.2 ± 13.4	66.5 ± 8.7
Scully PR, 2022^23^	24	82	NA	NA	NA	NA	23.8 ± 15	23.8 ± 15	NA	NA	NA	NA	NA	NA	NA	NA
Ishiyama M, 2023^24^	36	35	61	57	84.4 ± 5.2	83.8 ± 5.3	14.5 ± 7.8	14.5 ± 7.8	35.9 ± 5.02	29.8 ± 1.08	0.46 ± 0.15	0.46 ± 0.17	43.4 ± 15.1	46 ± 17.4	63.6 ± 12.5	65 ± 11.1
Vignale D, 2023^25^	29	84	38	57	82.6 ± 6.2	82.4 ± 3.8	13 ± 3	13 ± 3	34.6 ± 3.3	26.8 ± 3.0	0.44 ± 0.12	0.42 ± 0.1	41.4 ± 10.1	46.4 ± 9.8	56.6 ± 8.6	61.3 ± 5.3
Koike H, 2024^26^	150	150	44	46	80.7 ± 8.97	79.4 ± 9.3	12 ± 7.45	12 ± 7.45	32.2 ± 2.9	26.2 ± 1.6	0.40 ± 0.07	0.41 ± 0.1	38.9 ± 10	38.4 ± 10.6	55.4 ± 10.7	59.8 ± 8.4
Takahashi M, 2024^27^	44	83	55	64	85 ± 7	84 ± 4	12.9 ± 7.8	11.2 ± 6.1	35.9 ± 3.2	28.9 ± 2.2	0.42 ± 0.12	0.46 ± 0.11	51.3 ± 18	47.9 ± 15.4	55.3 ± 12.8	61.2 ± 9.1
Patel KP, 2024^28^	48	47	NA	NA	NA	NA	45.8 ± 15.2	45.8 ± 15.2	NA	NA	NA	NA	NA	NA	NA	NA

CT-ECV, computed tomography–derived extracellular volume; AVAi, indexed aortic valve area; LVEF, left ventricular ejection fraction; NA, not available.

**Table 4:** Comparison between high CT-ECV and low CT-ECV pooled groups obtained by the meta-analysis

	High CT-ECV pooled group (*n* = 524)	Normal CT-ECV pooled group (*n* = 699)	Effect size (SMD/OR)	*P*-value	Number of studies	Sample size
Age ± SD	82.34 ± 0.82	82.22 ± 0.71	0.053 ± 0.068	0.439	7	913
Female (%)	48.1	54.0	0.777	0.072	7	913
BMI ± SD	24.99 ± 1.25	25.36 ± 1.02	−0.116 ± 0.068	0.091	7	913
Creatinine ± SD	1.06 ± 0.08	0.99 ± 0.06	0.131 ± 0.092	0.152	5	500
BNP ± SD	732.19 ± 146.89	424.61 ± 85.73	0.625 ± 0.078	**<0.001**	5	729
HTN (%)	77.6	81.2	0.760	0.118	7	913
Diabetes (%)	34.9	25.5	1.605	**0**.**002**	7	913
Dyslipidaemia (%)	57.6	66.3	0.623	**0**.**005**	6	763
AF (%)	37.0	24.9	1.808	**<0.001**	6	800
Previous MI (%)	15.8	6.1	3.380	**0**.**001**	4	429
Previous PCI (%)	25.4	14.8	1.858	**0**.**017**	5	463
LVEF ± SD	52.5 ± 3.9	57.9 ± 2.6	−0.494 ± 0.065	**<0.001**	8	1022
Mean gradient ± SD	41.86 ± 3.70	44.7 ± 3.70	−0.142 ± 0.068	**0**.**039**	7	913
AVAi ± SD	0.41 ± 0.01	0.43 ± 0.01	−0.112 ± 0.068	0.100	7	913
LF-LG (%)	25.4	19.6	1.860	0.066	5	556
CT-ECV ± SD	36.84 ± 1.34	27.56 ± 0.77	2.486 ± 0.090	**<0.001**	7	913
AV calcium score ± SD	2387.23 ± 176.31	2445.47 ± 149.65	−0.073 ± 0.071	0.306	6	842

BMI, body mass index; BNP, brain natriuretic peptide; HTN, arterial hypertension; AF, atrial fibrillation; MI, myocardial infarction; PCI, percutaneous coronary intervention; LVEF, left ventricular ejection fraction; AVAi, aortic valve area index; LF-LG, low-flow, low-gradient aortic stenosis; CT-ECV, computed tomography–derived extracellular volume fraction; AV calcium score, aortic valve calcium score; SMD, standardized mean difference; OR, odds ratio. Bold when *P* < 0.05.

### Prognostic value of CT-ECV on cardiovascular outcomes after AVR

**Figure 2:**
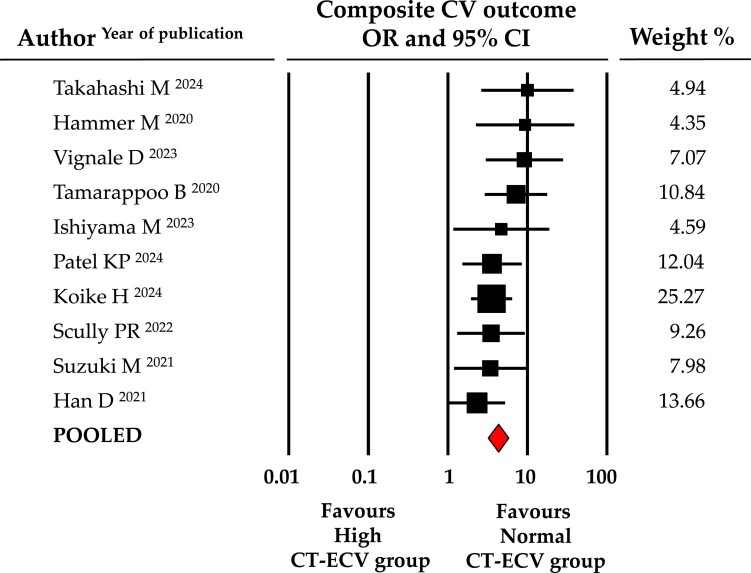
Forest plot for OR of the primary outcome (composite cardiovascular outcome) in patients with high vs. normal CT-ECV. Relative weight of each study is reported on the right side. CI, confidence interval; CV, cardiovascular, CT-ECV, computed tomography extracellular volume.

At a mean follow-up of 17.9 ± 2.3 months after AVR, patients with elevated CT-ECV (*n* = 524) experienced a significantly higher number of cardiovascular events (43.4% vs. 14.0%) compared with patients with normal CT-ECV (*n* = 699; *Graphical Abstract*). As shown in *Figure 2*, the primary cardiovascular outcome was four times more prevalent in the pooled high CT-ECV group than in the control group; with an OR being 4.3 (95% CI: 3.192–5.764, *P* < 0.001, data from 10 studies). The presence of a single study effect was excluded in the sensitivity analysis; a relevant publication bias was not present. The difference in event rates between the high CT-ECV and controls was still present after correction for publication bias (OR: 4.0, 95% CI: 2.975–5.236). Furthermore, given the substantial clinical divergence of the outcome utilized by Han *et al*. [recovery of LVEF^21^] compared with other included studies, a sensitivity analysis excluding it was performed. As shown in Supplementary data online, *Figure S1*, this analysis confirmed an almost five-fold higher risk of cardiovascular events in subjects with high CT-ECV (OR: 4.7, 95% CI: 3.437–6.493, *P* < 0.001).

**Figure 3:**
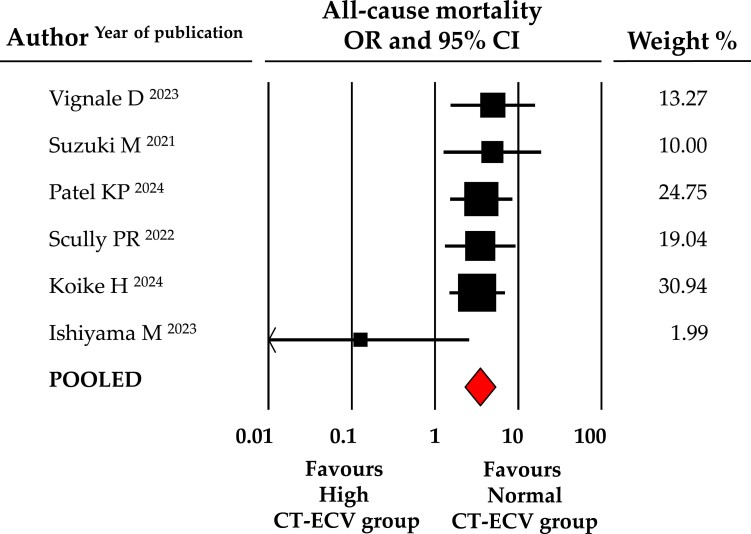
Forest plot for OR of all-cause mortality in patients with high vs. normal CT-ECV. Relative weight of each study is reported on the right side. CI, confidence interval; CT-ECV, computed tomography extracellular volume.

**Figure 4:**
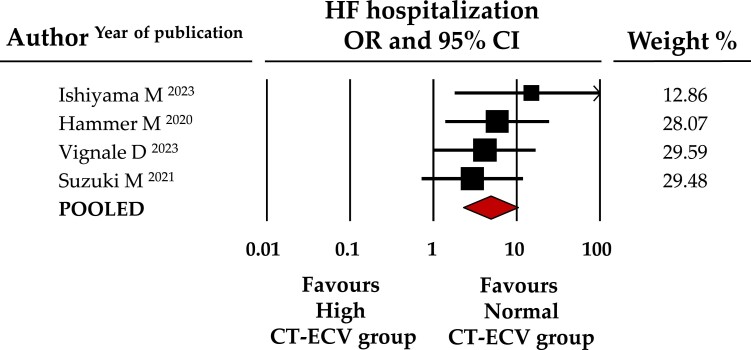
Forest plot for OR of HF hospitalizations in patients with high vs. normal CT-ECV. Relative weight of each study is reported on the right side. CI, confidence interval; HF, heart failure, CT-ECV, computed tomography extracellular volume.

Similarly, the occurrence of both secondary outcomes, all-cause mortality and HF hospitalization, was significantly higher in the high CT-ECV group. Specifically, all-cause mortality occurred in 29.3% of patients with elevated CT-ECV vs. 11.6% with CT-ECV below the cut-off (OR: 3.5, 95% CI: 2.276–5.311, *P* < 0.001, data from 6 studies, *Figure 3*), whereas HF hospitalization was observed in 25.5 vs. 5.9% (OR: 4.9, CI: 2.283–10.376, *P* < 0.001, data from 4 studies, *Figure 4*).

The negative prognostic value of CT-ECV in patients with severe AS undergoing AVR is further suggested by the meta-analysis of univariate regressions (expressed as hazard ratios: HRs) conducted across individual studies. Specifically, each percentage increase in CT-ECV corresponds to a 9% rise in the pooled relative risk of cardiovascular outcomes (pooled HR: 1.09, 95% CI: 1.064–1.111), whereas surpassing the CT-ECV cut-off elevates the relative risk by 320% (pooled HR: 3.2, 95% CI: 2.4–4.1).

### Correlation analyses

**Figure 5:**
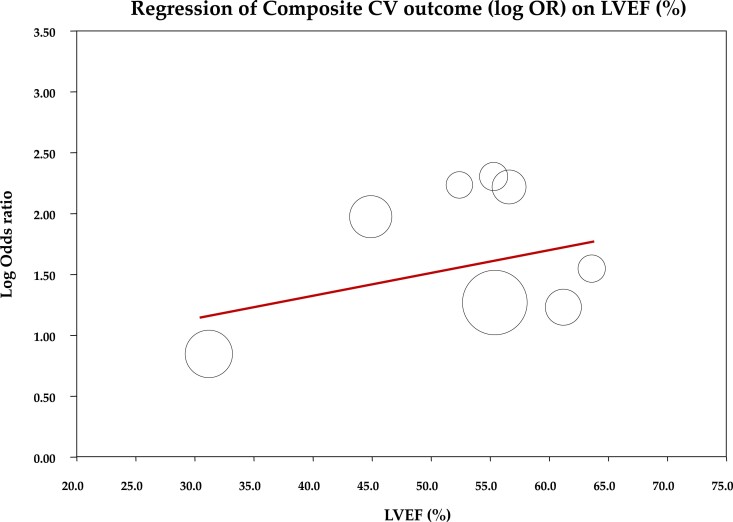
Meta-regression analysis between LVEF and the effect size of cardiovascular (CV) outcomes. OR, odds rartio.

Considering the more complex and severe clinical phenotype observed in the pooled high CT-ECV group (*Table 4*), with higher ECV cut-off values associated with an increased OR for the main outcome (coefficient: 0.1041, *P* = 0.043), it was hypothesized that the observed worse prognosis might be due to the presence of more severe clinical characteristics rather than solely to interstitial fibrosis detected by CT-ECV. Therefore, a meta-regression analysis was conducted between LVEF and the effect size of cardiovascular outcomes (expressed as OR), aiming to assess the impact of left ventricular function on outcomes in patients with high CT-ECV. The analysis illustrated in *Figure 5* did not reveal a significant relationship between OR of cardiovascular outcomes and LVEF (coefficient: 0.02, *P* = 0.27). Similarly, a history of previous MI did not correlate with the occurrence of cardiovascular outcomes in patients with high CT-ECV (coefficient: 3.12, *P* = 0.48). These findings suggest an independent prognostic value of CT-ECV on post-AVR prognosis.

Finally, we conducted a meta-regression analysis to explore potential factors contributing to heterogeneity among the included studies: sample size, publication year, post-contrast timing, and CT-ECV quantification method (mean vs. septal) did not significantly influence the effect size.

## Discussion

The main findings and clinical implications of the present meta-analysis are that (i) high CT-ECV independently predicts cardiovascular outcomes in patients with AS undergoing AVR and (ii) this new prognostic evaluation tool, based on CT-ECV, can be easily integrated into current AVR planning CT protocols. Kato *et al.*^30^ showed that CT-ECV was significantly elevated in patients with AS compared with controls and was even further elevated in patients with cardiac amyloidosis, underscoring its potential utility in differentiating between these pathologies. A recently published meta-analysis has revealed that certain CT-derived myocardial biomarkers play a significant prognostic role in patients with severe AS. Notably, CT-ECV and left ventricular global longitudinal strain are identified as strong predictors of adverse cardiovascular outcomes.^31^

CT-ECV quantification may require a small amount of additional contrast material due to the low contrast-to-noise ratio of late enhancement scans and the additional radiation dose needed for the extra scan. However, compared with CMR, CT-ECV offers shorter acquisition times, reduced susceptibility to artefacts, and is both faster and more cost-effective.^32^ Recent technological advancements in this field appear to be highly promising, offering exciting possibilities for future developments and applications.

In a recent single-centre study comparing photon counting detector (PCD)-CT with CMR for myocardial ECV quantification, PCD-CT demonstrated promising results in myocardial tissue characterization. Using both single-energy and dual-energy PCD-CT, ECV values strongly correlated with CMR reference measurements. Dual-energy PCD-CT, in particular, showed a robust correlation with CMR and, notably, achieved a 40% reduction in radiation dose compared with single-energy PCD-CT, making it a more efficient option.^33^ Mergen *et al.* evaluated dual-energy PCD-CT for ECV quantification in patients with severe AS, finding that this approach was both feasible and accurate for assessing myocardial tissue. Using iodine ratios from dual and single-energy late enhancement scans, they demonstrated a strong correlation between dual and single-energy-based ECV measurements, with minimal error and tight limits of agreement. Notably, dual-source PCD-CT allowed reliable ECV quantification at a low radiation dose, eliminating the need for a true non-enhanced scan, making it a valuable option for myocardial tissue characterization in this patient population.^34^

The management of patients with AS has significantly advanced over the past two decades, due to the widespread adoption of TAVR, valve-in-valve procedures, and advancements in surgical replacement protocols. However, defining the prognosis of patients eligible for AVR continues to be challenging. A Heart Team meeting is convened to evaluate patients in the ‘grey zone’ who have intermediate risk scores, taking into account clinical, anatomical and procedural factors, and not least the futility of intervention.

Whereas the most important measure in determining the need for AVR in AS is the degree of valvular obstruction, the myocardium is also subjected to progressive changes that may result in worsening cardiac performance and increasing morbidity and mortality.^35^ According to European Society of Cardiology guidelines about the management of valvular heart disease, myocardial fibrosis is an important prognostic parameter in the setting of AS and its myocardial extension a major driver of left ventricular deterioration.^36–38^

CMR is the primary imaging modality for myocardial tissue characterization. CMR parametric mapping now allows routine spatial visualization of quantitative changes in the myocardium based on alterations in myocardial parameters T1, T2, T2*, and ECV.^39^ However, the use of CMR during clinical practice is limited due to its expensive and time-consuming nature. Instead, CCTA is an imaging tool that is mandatory in pre-procedural planning before TAVR or SAVR, and CT-ECV acquisition could stratify a patient’s cardiovascular risk in a short time and with a single examination. Recently, the meta-analysis by Kato *et al*. not only demonstrated a strong correlation between CT-ECV and ECV derived from CMR but also revealed that individuals with AS exhibit higher CT-ECV values compared with healthy controls, though lower than those seen in patients with cardiac amyloidosis.^30^ The results of our study support the prognostic relevance of CT-ECV in patients with severe AS undergoing AVR and suggest the use of this novel marker detected by CCTA in assessing prognosis.

### Baseline characteristics of the populations

As previously illustrated, the characteristics of the included studies revealed a heterogeneous population, with studies conducted across three different continental areas and employing different CT methodologies. Patients with low-flow, low-gradient AS were considered in the analysis, although they usually have a higher risk of adverse outcomes.^40,41^ CMR revealed more adverse left ventricular remodelling, higher ECV, and higher late gadolinium enhancement in these patients than in ‘classic high-gradient AS’. The extension of fibrosis is associated with a high risk of cardiovascular mortality^42,43^; there are few data from different CMR studies, which demonstrated the correlation between myocardial ECV and cardiovascular outcomes.^42,44^ Despite the heterogeneity of the population, the quality assessment of the studies indicates fair to good quality overall, ensuring confidence in the reliability of the findings. Moreover, the relevance of these findings in such a heterogeneous population lies in their broader applicability to real-world settings. Notably, the majority of studies focused on patients undergoing TAVR, reflecting the growing prominence of this minimally invasive procedure. Accordingly, it is essential to emphasize that CT-ECV evaluation does not require additional administration of contrast agents, implying only an extra scanning of the heart, thus eliminating any additional risks associated with this method.

### High CT-ECV and cardiovascular outcomes in patients with AS undergoing AVR

Our analysis revealed a significant difference in cardiovascular outcomes between patients with elevated CT-ECV values and those with normal values. This result expands the findings from previous CMR studies, which demonstrated that increased ECV represents a marker of left ventricular dysfunction and a powerful predictor of mortality.^38,45^ This could suggest the potential benefit of CT-ECV to accurately predict cardiovascular outcomes for this high-risk population. Patients with elevated CT-ECV experienced a substantially higher prevalence of cardiovascular events, including all-cause mortality and hospitalization due to HF, compared with those with normal CT-ECV. This finding underscores the prognostic value of CT-ECV as a predictor of adverse cardiovascular outcomes following AVR in patients with severe AS.

The observed association between elevated CT-ECV and worse cardiovascular outcomes remained robust across sensitivity analyses and after adjusting for potential publication bias. Furthermore, the conducted meta-regression analysis did not find a significant relationship between LVEF and cardiovascular outcomes in patients with high CT-ECV, suggesting that the adverse prognostic impact of elevated CT-ECV is independent of left ventricular function.

### Clinical implications

The clinical implications of our findings are significant. CT-ECV is a new, promising tool, easy to use, with wider availability, especially for patients who cannot perform a CMR. Moreover, CT-ECV is derived from the standard pre-AVR protocol; therefore, it is an ideal tool, being non-invasive, with no additional financial costs aside from the time required for a post-contrast acquisition and without any significant supplementary risks. The worse prognosis in high CT-ECV patients strongly endorses the potential benefit of using this method to identify patients at higher risk of adverse cardiovascular events following AVR. This information could aid clinicians in risk stratification and treatment decision-making, potentially guiding the selection of optimal management strategies and finally improving patient outcomes. Should a CT-ECV cut-off associated with futility of the procedure be established, it would enable the avoidance of unnecessary and costly AVR procedures. Additionally, CT-ECV could also identify those patients who could benefit from more aggressive post-intervention follow-up. Many questions remain unanswered: How does CT-ECV change over time in patients? Does it increase, decrease, or is it modified by AVR? Could the detection of elevated CT-ECV in patients with moderate AS become a parameter suggesting early intervention in the future?

The findings of our study highlight the complex nature of CT-ECV as a prognostic marker in severe AS, emphasizing the need for standardized protocols that can more consistently apply ECV as a prognostic measure in clinical practice.

## Limitations

Our meta-analysis has several limitations that we would like to address. One of the primary constraints of our study pertains to the inclusion of studies with heterogeneous cardiovascular outcomes, albeit closely aligned, except for the study by Han *et al.*, which diverges clinically due to its focus on LVEF recovery. To mitigate this limitation, as delineated in the Results section, we undertook sensitivity analyses (excluding the study by Han *et al.*) and secondary analyses to assess individual outcomes, specifically all-cause mortality and HF hospitalization.

Furthermore, the included studies utilized different CT scanners and acquisition modalities, slightly varied acquisition times and contrast doses, and heterogeneous methodologies to determine CT-ECV; these technical differences (outlined in *Table 2* and Supplementary data online, *Table S1*) and the lack of standardized protocols may affect consistency across studies and have consequently influenced our findings. Especially, the stability of Hounsfield units is crucial for accurate quantification, yet CT-ECV faces lower signal-to-noise ratios than CMR, particularly affecting late enhancement scans.^32^ Hence, current and future research should prioritize optimizing contrast protocols to improve the contrast-to-noise ratio.^46^

Moreover, as previously mentioned, only two studies (Scully *et al.*^23^ and Patel *et al.*^28^) specifically screened and excluded subjects with cardiac amyloidosis, the presence of which could reasonably elevate CT-ECV values, as demonstrated by Shingo *et al.*,^30^ justifying a worse prognosis in the setting of severe AS. Actually, our findings reflect real-world scenarios, as it is not common practice to routinely screen for cardiac amyloidosis in all patients with AS. To address this limitation, we intended to conduct a meta-analytical comparison of myocardial mass between the two groups, hypothesizing that significantly higher mass values in the high CT-ECV group might indicate a higher prevalence of subjects with cardiac amyloidosis. Similarly, we planned to perform a meta-regression assuming that as mass values increased, prognosis would worsen. Regrettably, myocardial mass data were reported heterogeneously across studies (LVM or indexed LVM, measured by echocardiography or CMR), thus precluding the possibility of conducting analyses on more than three studies.

Similarly, not all the studies accounted for and excluded any infarcted component in the evaluation of CT-ECV. Unlike CMR, which provides precise visualization of infarcted areas through late gadolinium enhancement, CT may not adequately identify these regions. This limitation can result in the inclusion of infarcted myocardium in ECV measurements, leading to artificially elevated CT-ECV values, as the assessment may include both healthy and infarcted myocardial tissue. A history of previous MI can reasonably both increase CT-ECV values and has an independent prognostic impact. To address this, we conducted a meta-regression, which found that a history of previous MI did not correlate with the occurrence of cardiovascular outcomes in patients with high CT-ECV. Caution is warranted when interpreting ECV values derived from CT in infarcted patients, as they may not fully reflect the underlying pathophysiology.

Finally, since a patient-level meta-analysis was not conducted, it was not possible to identify a CT-ECV cut-off that accurately predicts post-AVR prognosis. As of now, there is no officially recognized cut-off, and each study has determined its own, albeit using different methods (see *Table 2*). To address this limitation, we decided to provide a pooled cut-off value based on those used in the various studies, believing that its determination (along with its CI) may still hold clinical significance. Of course, our choice can be debated, given the heterogeneity in methods used by the different studies to establish their cut-offs (ROC curve, median, third tertile) and the variability in the values found (e.g. individual study cut-offs ranged widely from 27.1% to 40.8%), introducing a degree of imprecision that may limit its immediate clinical applicability. This variability underscores the need for further research to validate an optimal, standardized cut-off threshold, which could improve the clinical utility and prognostic value of CT-ECV. Nevertheless, we believe that the pooled value was the only data we could produce, and it still represents a ‘reasonable’ value.

## Conclusions

This meta-analysis demonstrates that elevated myocardial CT-ECV is independently associated with adverse cardiovascular outcomes in patients with severe AS undergoing AVR. CT-ECV assessment does not require additional contrast use or financial costs. This underscores its potential as a practical and cost-effective prognostic tool for risk stratification. The findings suggest that CT-ECV evaluation could aid clinicians in identifying high-risk patients and guiding treatment decisions, ultimately improving patient outcomes. The implementation of CT-ECV evaluation in routine AVR planning protocols should be considered. Further research is warranted to definitely validate and refine the utility of CT-ECV in this setting.

## Supplementary data

Supplementary data are available at *European Heart Journal - Cardiovascular Imaging* online.

## Supplementary Material

jeae324_Supplementary_Data

## Data Availability

The data underlying this article will be shared on reasonable request with the corresponding author.
