# Supplementary material for: Prognostic value of myocardial computed tomography–derived extracellular volume in severe aortic stenosis requiring aortic valve replacement: a systematic review and meta-analysis
Source: Eur Heart J Cardiovasc Imaging. 2025 Jan 10;26(3):518–31. doi: 10.1093/ehjci/jeae324 (PMC11879236; doi:10.1093/ehjci/jeae324)
Supplement: jeae324_Supplementary_Data [file jeae324_supplementary_data.zip › def_Supplementary_Composite outcome forest plot NO HAN .pptx]

## Slide 1
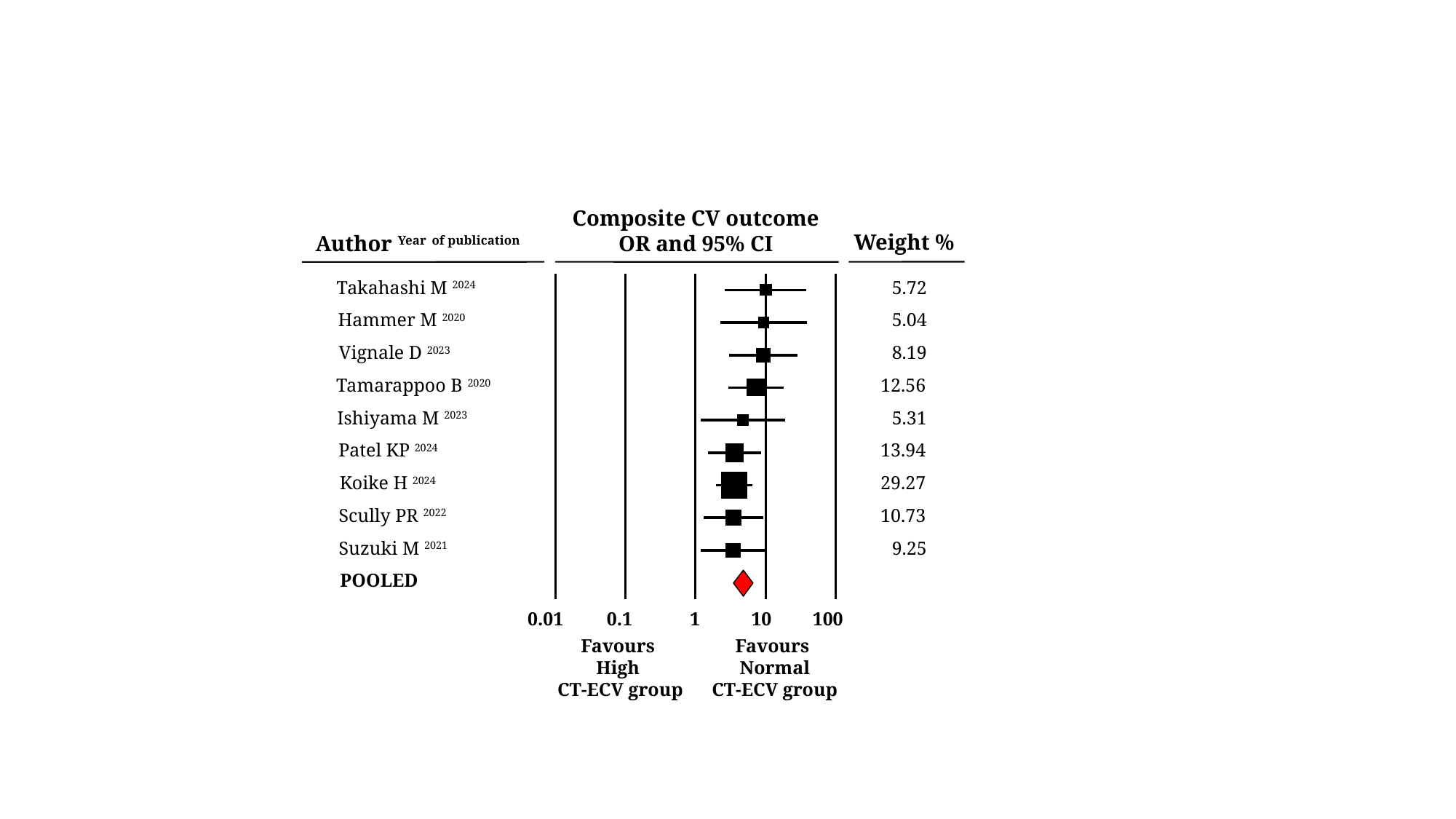

Composite CV outcome
OR and 95% CI
Weight %
Author Year of publication
Takahashi M 2024
5.72
Hammer M 2020
5.04
Vignale D 2023
8.19
Tamarappoo B 2020
12.56
Ishiyama M 2023
5.31
Patel KP 2024
13.94
Koike H 2024
29.27
Scully PR 2022
10.73
Suzuki M 2021
9.25
POOLED
0.01
0.1
1
10
100
Favours
High
CT-ECV group
Favours
Normal
CT-ECV group
