# Supplementary material for: Prognostic value of myocardial computed tomography–derived extracellular volume in severe aortic stenosis requiring aortic valve replacement: a systematic review and meta-analysis
Source: Eur Heart J Cardiovasc Imaging. 2025 Jan 10;26(3):518–31. doi: 10.1093/ehjci/jeae324 (PMC11879236; doi:10.1093/ehjci/jeae324)
Supplement: jeae324_Supplementary_Data [file jeae324_supplementary_data.zip › Supplementary table 1.docx]

**Supplementary table 1: Contrast media protocols protocols of the studies included in the systematic review and meta-analysis**

| **Author, Publication year** | **Contrast Media (CM) Protocol** |
| --- | --- |
| **Hammer M**  2020 ]15] | 50–60 ml IV injection of nonionic CM (Iopromide 370, Bayer Shering, Berlin, Germany), flow rate of 3 ml/s |
| **Tamarappoo B**  2020 [20] | 100 ml IV bolus injection of iodine CM, Omnipaque (GE Healthcare, Little Chalfont, UK) |
| **Han D**  2021 [21] | 100 mL IV bolus injection of iodine CM, Omnipaque (GE Healthcare, Little Chalfont, UK) |
| **Suzuki M**  2021 [22] | 12 mL of CM (Iopamiron 370, 370 mg/mL; Bayer Yakuhin, Osaka, Japan) to assess the CM transit time, using 0.6 mL/kg of CM at a flow rate of 0.06 mL/kg per second, followed by 0.6 mL/kg diluted CM (1:1; contrast/ saline) at the same flow rate.  Mean CM: 0.93±0.26 mL/ kg. |
| **Scully PR**  2022 [23] | 90 ml (including the 10 ml timing bolus) IV bolus injection of iodine CM, Omnipaque 300 (GE Healthcare, Chicago, Illinois) |
| **Ishiyama M**  2023 [24] | 0.84 ml/kg IV injection over 12 s (26 mgI/kg/s) of CM |
| **Vignale D**  2023 [25] | IV CM bolus tailored to patients’ size [85 mL for body mass index (BMI) <25; 95 mL for BMI 25–30; and 110 mL for BMI >30]. Use of iodinated CM, Visipaque 320 (GE Healthcare, Little Chalfont, Buckinghamshire, UK). |
| **Koike H**  2024 [26] | IV bolus injection of CM (Omnipaque-350, GE Healthcare Inc), averaging a total of 100 ± 30 mL (adjusted depending on the patient’s renal function, body mass index, and kV used for cardiac imaging) |
| **Takahashi M**  2024 [27] | IV bolus 40–100mL of undiluted iodinate CM (350–370mgI/mL) at 3–5mL/s, followed by 0–50mL of a 50%/50% saline-to-CM mixture at 3–4mL/s and 20–30mL of pure saline at 2–4mL/s |
| **Patel KP**  2024 [28] | 90 ml (including the 10 ml timing bolus) IV bolus injection of iodine CM, Omnipaque 300 (GE Healthcare, Chicago, Illinois) |
